# Red Raspberry (*Rubus idaeus* L.) Seed Oil: A Review

**DOI:** 10.3390/plants10050944

**Published:** 2021-05-09

**Authors:** Audronė Ispiryan, Jonas Viškelis, Pranas Viškelis

**Affiliations:** Lithuanian Research Centre for Agriculture and Forestry, Institute of Horticulture, Kauno str. 30, Kaunas District, LT-54333 Babtai, Lithuania; jonas.viskelis@lammc.lt (J.V.); pranas.viskelis@lammc.lt (P.V.)

**Keywords:** bioactive compounds, red raspberry, nutritional value, seed oil

## Abstract

Raspberry (*Rubus idaeus* L.) seed oil (RSO) is considered as a source of high value bioactive compounds as fatty acids, tocopherols, tocotrienols, carotenoids, flavonoids, phytosterols, antioxidants, monoterpenes and many other chemical constituents. These compounds are appreciated as a source of nutrition for humans, as additives in cosmetic production, has immense therapeutic potential. Raspberry seed oil exerts many pharmacological effects included antimicrobial, antioxidant, anti-inflammatory activity and many other effects. The various databases like PubMed and Science Direct were used to identify, analyze and summarize the research literature on raspberries. This review will highlight recent developments of the chemical constituents and nutraceutical and cosmetical effects of RSO. Practical application: analyzed recent researches and international patents containing raspberry seed oil can help practitioners of various industries create new high-value products.

## 1. Introduction

Recently has been observed a considerable openness and greater attention of researchers concerning the characterization of essential oils and secondary metabolites on known and lesser known plants which have highlighted how natural resources on this particular issue can still provide new scientific data scientific data which can be useful for human health [1,2]. Berries are a valuable source of a wide range of secondary metabolites, which can be used in pharmaceutical, agronomic and food industries [3,4,5,6].

In 2019, world production of raspberries was 822.49 K metric tons (mt), with Russia as the leading producer, supplying 22.0% of the world total. Other major producers were Mexico (16.3%), Serbia (15.2%), United States (13%) and Poland (9.6%) [7]. Most of raspberries are used for processing juices, jams, wine, etc. [4]. During the processing a large amount of berry by-products (pomace, seeds, etc.) are produced. Raspberry seeds are considered as by-product or waste [8]. Oil from raspberries seeds is receiving increasing attention among scientists, farmers, processors and consumers. This kind of berry oils often have a unique fatty acid profiles and has interesting other minor bioactive components that are in demand on the market [9].

Furthermore, raspberry seed oil (RSO) production provides the use of a renewable resource, adding value to agricultural products and improving the environment. Raspberry seeds contain up to 12.2% protein and has 11–23% oil. These oils have unique characteristics that makes interest to the cosmetics and medical industries [10]. The fruit and berry oils are characterized by gentle processing (no refining, cold pressing, etc.), unique aroma and health-promoting attributes, low production yield and high price [11].

Food industry is interested in creating more added value products through the applications of by-products [12]. Berry seed oils are often considered to be specialty oils with unique bioactive components [13]. These kinds of oils also have the most valuable plant fats [14]. Due to the various bioactive compounds, they can be included in functional foods [15], are appreciated as a diet component and preventing the development of various diseases [16].

The main aim of the work was to analyze the quantitative and qualitative characteristics of raspberry seeds oil. This review also proposes an overview of the possible RSO processing technologies and quality factors, pros and cons, significance of RSO for human health, nutrition and cosmetical value. Starting from this, an analysis of the most recent international patents related to RSO was carried out, in order to provide farmers, company managers, customers and other stakeholders an insight into the most suitable solution for the economic and environmental sustainability of the raspberry seeds oil in management chain.

## 2. Raspberry Seeds Oil Processing Technologies and Quality Factors

The quality of fruit seed oil highly depends on fruit genotype, growing region and conditions, seed pretreatment, drying parameters and oil extraction techniques. These factors significantly affect the extracted oil yield, it’s bioactive compounds, antioxidant activity and oxidative stability [17].

The bioactive compounds of by-products can be extracted with Soxhlet extraction, hydro-distillation, maceration, cold-pressing and supercritical fluid extraction (SFE). SFE utilizes solvents (such as carbon dioxide) in its supercritical state, in which the solvent acts simultaneously as a liquid and as a gas, resulting in a more efficient extraction process [18].

Cold-pressing and SFE are the green methods for the extraction of valuable compounds from berry seeds, it does not use hazardous organic solvents. The only drawback of these green methods is that higher extraction yields are obtained with solvent extraction than SFE [19]. The advantages of working with SFE and cold-pressing are reduced solvent use, lower energy consumption, shorter extraction time and better quality [20]. There is still a need to utilize more novel and green techniques to the waste materials to achieve higher biologically active compounds retrieval rates.

## 3. Physico-Chemical Characterization

Raspberry seed oil is slightly cloudy, yellowish in color. This yellowish tinge to the oils is given by carotenoids. A yellowish tint is desirable because it gives the oil the characteristic butter-like appearance specifically to the oil without the addition of conventional dyes that are often used in the food industry [21].

Oil yield from the seeds is 10–23% [6,16]. Raspberry seed oil has a high content of *n*-6 and *n*-3 essential fatty acids and is an important qualitative characteristic of an oil. The acidic value of raspberry seed oil is ranging from 17.18 to 18.74 mg KOH g^−1^. The increasing acidity can be due to longer storage time of the berries [21].

The most unsaturated fatty acids in RSE are linoleic acid, a-linolenic acid and oleic acid. RSE if a good nutrient due to high content (78.9–85.5%) of polyunsaturated fatty acids and phytosterols (5384.1 μg g^−1^) [6,16].

Fatty acids are vital components of human diet and are required by cells of the body in the form of phospholipids for structural membrane integrity [22]. The content of polyunsaturated fatty acids in red raspberry seed oil was reported as of 85%, of which (as percentage of total fatty acids) had 54% of linoleic acid and 32% of α-linolenic acid.

In RSO there are also polyphenol compounds (2.65 mg 100 g^−1^), phytosterols (5.38 mg g^−1^), including campesterol, stigmasterol, sitosterol, avenasterol, cytrostadienol; and carotenoids, including zeaxanthin, β-carotene, lutein and cryptoxanthin [23]. RSO also contains large amounts of vitamin E (301.9 mg 100 g^−1^); tocopherols (295.19 mg 100 g^−1^), including α-tocopherol (71 mg 100 g^−1^), γ-tocopherol (272 mg 100g^−1^), Δ-tocopherol (17.4 mg 100 g^−1^); and tocotrienols (6.73 mg 100 g^−1^) [23,24].

Antioxidants are another very important dietary components, including vitamin E, phenolic compounds and tocopherols, which protects the body from free radical damage among other functions. These components are prevalent at significant levels in raspberry seed oils [25,26].

Summarizing the research literature, RSO is a unique source of bioactive phytochemicals containing high levels of antioxidant components. Oil yield from the raspberry seed is not high (from 10 to 20%). RSO well-known for its components: fatty acids, vitamin E so appreciated in cosmetics and pharmaceuticals industries. RSO is also known for its high antioxidant capacity and exhibits anti-inflammatory, anti-mutagenic and antimicrobial properties [27]. RSO is also used in cosmetics as an efficient moisturizer and emollient which helps to reduce the oxidative stress in skin, is used in cosmetic emulsions for UV protection [6].

## 4. Significance of RSO for Human Health

### 4.1. Nutritional Value

Safe and natural medicinal foods are gaining significance in mainstream healthcare [28,29,30]. An increasing number of studies in recent years have shown the health benefits of using raspberry seed oil as a dietary supplement in the global scientific literature. The main ingredients of RSO are essential fatty acids (C18:2 *n*-6, linoleic and C18:3, *n*-3, α-linolenic acid) with a 1.8-fold prevalence of linoleic acid [26,31,32]. The human organism cannot synthesize them, but they are required for a good health [33]. The ratio of omega-6 to omega-3 fatty acids in the RSO is very favorable for nutrition (approximately 1.4:1).

According to the European Scientific Committee on Food (ESCF), 2% of the total daily energy intake should be derived from omega-6 and 0.5% from omega-3 polyunsaturated fatty acids [34], which corresponds to a daily intake of approx. 6 g per day for woman (5 g of omega-6 and 1 g of omega-3) and 8 g per day for men (6.4 g of omega-6 and 1.6 g of omega-3) [35]. A diet rich in fatty acids and low in dietary fiber increases the risk of obesity, type 2 diabetes, cardiovascular and many other diseases [36]. Meanwhile the World Health Organization (WHO) recommends a 2.5–9% of omega-6 intake and 0.5–2% of omega-3 fatty acid intake of daily energy. These differences between ESCF and WHO recommendations occurs due to different nutritional goals, where ESCF recommendation is based on the amounts necessary to correct a clinically overt deficiency, WHO recommendation is based on considerations of cardiovascular health and neurodevelopment [37]. Due to the presence of fatty acids in raspberry seed oil, it can be used as a dietary supplement and has a positive effect against many diseases [38].

The RSO is also rich in tocopherols—3.3–3.5 mg g^−1^ (compounds with vitamin E activity). A health claim has also been confirmed for this vitamin: “Vitamin E helps protect cells from oxidative damage”. The recommended daily allowance for vitamin E is from 4 mg for children to 15 mg for adults. This means that 3–5 g of oil per day fully supplies the human body with vitamin E, which helps to protect cells from the damage caused by free radicals, boosts immune system, helps to keep blood from clotting with blood vessels, might help to prevent Alzheimer’s disease, maintain brain health and carry out many other important functions [39,40,41].

### 4.2. Cosmetical Value

RSO is gaining increasing attention by cosmetics industry. It is used as an ingredient in body and face moisturizers because of its high concentrations of Vitamins A and E. These vitamins are essential for the maintenance and repair of skin cells. The oil works by creating a lipid barrier that stops skin from losing natural moisture. Retaining moisture helps to keep skin cells looking young and full. Raspberry oil can be used as a base for makeup applications. It adds adding hydration, sun protection and nourishing vitamins.

The primary factors that contribute to premature aging of the skin include UV from the sun, illness, smoking and drinking. Raspberry seed oil is packed with carotenoids—a plant derived source of Vitamins A and E. These compounds are widely used in many anti-aging skin care products to help promote youthful skin [25,42]. Vitamin A is a popular antioxidant and ingredient in anti-aging skincare products because it adds moisture, reduces the appearance of wrinkles and smooths skin texture. Vitamin E is another highly praised antioxidant in the anti-aging industry. It helps to protect cells from oxidative damage and assists with maintaining collagen structure [43].

Research has demonstrated that people with higher levels of antioxidants have fewer and less pronounced wrinkles than those with low levels. This oil is of particular interest to medical experts (and us natural product enthusiasts) because it naturally contains sun protective compounds in addition to its beneficial antioxidants [42].

Raspberry seed oil is a very lightweight gentle moisturizing solution. Unlike other emollients, it does not clog pores and encourages natural water retention in the cells. This keeps them looking full, giving a more youthful appearance and reduces the appearance of fine lines and wrinkles. RSO is also noncomedogenic, meaning it will not clog your pores. Use it to moisture your face without blocking your pores [44]. Additionally, raspberry oil’s sun protective qualities offer added benefit to people looking for a mild, non-irritating moisturizer with a sun protection factor (SPF) [45,46].

Raspberries contain antimicrobial properties that are powerful enough to stop the growth of harmful bacteria such strains such as salmonella and *E. coli* (*Escherichia coli*). Although there is no substitute for proper oral hygiene, raspberry seed oil might be beneficial in destroying harmful bacteria found in the mouth. It might also assist in healing painful and inflamed gums that have been irritated by the plaque deposits [47,48]. RSO can also moisturize and soften the skin as well as reduce skin irritations such as itching, swelling and redness [49,50].

Oomah et al. [6] reports that RSO can be used as a broad-spectrum UV protectant and provide protection against both UV-A and UV-B. However, not many other SPF tests on raspberry seed oil have been made, but the interest in RSO has accelerated [51]. Meanwhile, a very recent research by Ácsová et al. [49] in 2021 has revealed that the oil may not be as effective as concluded in Oomah et al. [6] research. In the latest study SPF values of the RSO in vitro was 0.4, in vivo 2.6, and it is significantly lower than the values reported in the controversial studies. Ácsová et al. [49] showed that the overestimated SPF values of RSO was determined by authors who did not strictly followed Mansur’s original methodology.

It is sure that RSO can make a great addition to an organic product because of its abundant amount of antioxidants, including Vitamin E, which helps to block free radicals. Not to mention plenty of the incredibly beneficial micronutrients called polyphenols. Therefore, with the growing demand for natural sunscreen products, it would be useful to conduct in-depth research to substantiate or refute one or another author.

## 5. Patents on RSO

When an inventor finds a solution to a particular problem, one needs to make sure the solution is new. In addition, the description of the invention must indicate and compare solutions to similar problems with the patented solution. Determining the state of the art makes it possible to see which technical field is already protected by patents and to predict the direction in which new solutions can be sought. The search for raspberry seeds oil novelty and technical level (also called patent search) was carried out in publicly available free international patent databases containing data on issued patents [52]. Table 1 illustrates an updated report on application of RSO.

The oldest registered patent relating to raspberry seed oil is in 1975 in France. The invention relates to cosmetic or pharmaceutical compositions, more particularly products for dental care, skin creams and lotions, shampoos and make-up. One frequently meets in cosmetology phenomena of inflammation, such as that of the gums, called gingivitis, that of the epidermis, called erythema, eczemas or other skin lesions. The origin of these inflammations can be very varied: biological deficiency, allergy, the effect of the sun’s rays and often the ingredients of the beauty products themselves.

Substances with an anti-inflammatory effect are well known, such as cortisones, phenylbutazone, salicylates, indomethacin, anthranilic acid derivatives, proteases, which, besides their effectiveness, also cause side effects. The subject of the invention is the incorporation into cosmetic or pharmaceutical products of a new anti-inflammatory substance of natural origin, pressure oil or raspberry seed extraction oil, hereinafter called raspberry seed oil. Raspberry seeds, capable of preventing or suppressing inflammatory phenomena, for example of the gums or the epidermis.

The patent authors state that RSO, expressed or extracted from raspberry seeds have anti-inflammatory activity and are useful in anti-sunburn preparations, dental preparations, mouth-washes, after shaving preparations, antiperspirants, shampoos, lipsticks, etc. RSO as a dietary supplement has multiple functions of lowering blood lipid, cholesterol and blood pressure, resisting thrombus and arteriosclerosis, preventing cardiovascular disease, enhancing memory and preventing Alzheimer’s disease and cancer and has a high nutritional value and health care function. The authors also note that changing waste (raspberry seeds) into high value products having great significance for the development of the red raspberry industry and the comprehensive utilization of byproducts.

**Figure 1:**
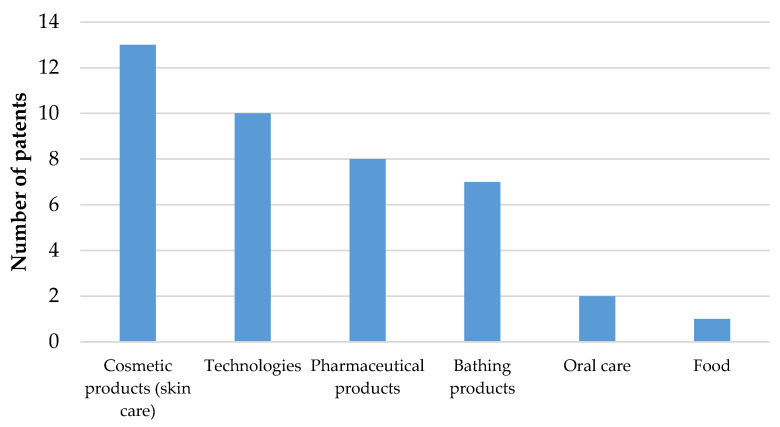
International patents relating to raspberry seeds oil.

The countries that have registered the most patents are China (17 patents) and USA (11 patents). It can be concluded that the fatty acids, vitamins A and E help in resorting skin elasticity, skin hydration, thus, finding its implication as anti-aging and in various other skin diseases as a result, oil has found a large niche in the cosmetics industry and is also significant in the pharmaceutical industry (Figure 1).

## 6. Conclusions

Raspberry seeds oil can be used in food, pharmaceutical, cosmetic and chemical industries, it has a medicinal and therapeutic value. The so-called green oil production methods (SFE and cold-pressed techniques) ensure the sustainable realization of high-value products that meet the needs of the consumer of this time, the development of zero-waste technology in the circular economy. RSO has high content of polyunsaturated fatty acids, tocopherols, polyphenols and fatty acids which help in the prevention and treatment of various disorders. The benefits of RSO for external use have been extensively studied, products are widespread and recognized by consumers in the cosmetics industry. However, there is a lack of information on RSO as food consumption.

To conclude this review, RSO represents a potential source of natural ingredients for food, cosmetics and pharmaceutical industries. Due to the high nutritional value of raspberry by-products, it can be exploited as food additives or supplements providing the high valuable products which may be economically attractive for consumers. Both internal and external consumption of raspberry seed oil have a significant impact on human health, but there is a lack of data on internal consumption dosing and treatment for the prevention of specific diseases.

In the future, it would be useful to examine the influence of the oil on the internal consumption. It would be appropriate and interesting to confirm or deny the properties of the oil for sun protection as well. There is also a lack of information on the impact of different oil extraction technologies on its yield and quality, which would be valuable in practice for business representatives.

## Figures and Tables

**Table 1:** Updated list on patents of raspberry seeds oil.

Patentscope	Patent’s Title	Publication Number	Publication Year and Office
1. Technologies	1.1. Dimethicone copolyol raspberriate as a delivery system for natural antioxidants;	6630180	2003, USA
	1.2. Raspberry amido amines and betaines as a delivery system for natural antioxidants;	7078545	2006, USA
	1.3. Synergistic super potent antioxidant cold pressed botanic oil blends;	20070243310	2007, USA
	1.4. Method of making edible oil with unsaturated fatty acid content of more than 90% by extracting roasted bramble seed with hexane purpose;	1020070080027	2007, Korea
	1.5. Immune enhancement by seed oil;	20090324759	2009, USA
	1.6. Ultrasonic wave auxiliary extraction method for extracting raspberry seed oil;	102864012	2013, China
	1.7. Composite extract of black raspberry oil, raspberry oil and mulberry oil;	WO/2015/137633	2015, Korea
	1.8. Preparation method of raspberry seed oil and product prepared therefrom;	106947583	2017, China
	1.9. Raspberry seed oil extraction technology;	109022136	2018, China
2. Pharmaceutical products	2.1. Raspberry seed oil compsns—with antiinflammatory activity, for cosmetic and pharmaceutical use;	2255055	1975, France
	2.2. Topical steroid spray with botanic seed oils;	20090304603	2009, USA
	2.3. Berry oils and products;	20110280971	2011, USA
	2.4. Raspberry seed oil soft capsule and preparation method thereof;	102687861	2012, China
	2.5. Dietary supplement to treat dry eyes;	2013101038	2013, Australia
	2.6. Traditional chinese medicine essential oil for relieving fatigue and preparation method of traditional chinese medicine essential oil;	104800783	2015, China
	2.7. Complex extract of black raspberry oil, raspberry oil and mulberry oil;	1020160047055	2016, Korea
	2.8. Soft-capsules containing sea buckthorn seed oil;	108497499	2018, China
3. Cosmetic products	3.1. Cleansing sheet;	2003226637	2003, Japan
	3.2. Compositions, to reinforce and restore functional barrier of skin and to control inflammation, comprises insaponifiable fraction of rape oil;	2912652	2009, France
	3.3. Skin care compositions with botanic seed oils;	20090123578	2011, USA
	3.4. Natural korean herb cosmetics capable of being applied to sensitive skin;	101262557	2013, Korea
	3.5. Anti-aging cosmetic composition;	WO/2013/066623	2013, USA
	3.6. Cream pack containing raspberry;	106955249	2017, China
	3.7. Eye cream containing raspberry;	107041862	2017, China
	3.8. Healthcare chest-enlarging weight-losing molding multifunctional massage oil for external use and preparation method thereof;	106667859	2017, China
	3.9. Novel herbal sunscreen formulation and method thereof;	201611003234	2017, India
	3.10. Anti-aging, soothing and moisturizing gel and preparation method thereof;	108852928	2018, China
	3.11. Environmental-protection facial mask base material and preparation method and application thereof;	108926504	2018, China
	3.12. Anti-wrinkle oil-control acne-removing repair mask and preparation method thereof;	108904371	2018, China
	3.13. Sun-protection lipstick and preparation method thereof;	111789782	2020, China
4. Bathing products	4.1. Dandruff treatment compositions with anti-inflammatory agents including botanic seed oils;	20090317502	2009, USA
	4.2. Raspberry soap composition having antibiotic and antioxidant function by comprising raspberry seed oil and raspberry wine;	1020120052467	2012, Korea
	4.3. Cosmetic or bathing product containing rubi fructus seed extract and antioxidative ingredients purpose;	1020110129606	2012, Korea
	4.4. Silicon oil free shampoo capable of preventing alopecia and preparation method of shampoo;	106473994	2017, China
	4.5. Anti-soap formulation;	20190133921	2019, USA
	4.6. Preparation method of de-oil shampoo;	109925228	2019, China
	4.7. Oil-control shampoo;	109925231	2019, China
5. Oral care	5.1. Novel composition for herbal mouthwash and process for the preparation of the same;	1376/DEL/2012	2014, India
	5.2. Whitening and anti-sensitivity aloe gel and preparation method thereof;	108714130	2018, China
6. Food	6.1. fruit products containing omega-3 fatty acids.	WO/2010/011712	2010, USA

## Data Availability

Not applicable.
